# A Hyperflexion Hallux Mallet Injury: A Case Report

**DOI:** 10.5704/MOJ.2207.016

**Published:** 2022-07

**Authors:** GKY Tan, MSJ Chew, S Sajeev, A Vellasamy

**Affiliations:** 1 Department of Medicine, Lee Kong Chian School of Medicine, Singapore; 2 Department of Orthopaedic Surgery, National University of Singapore, Singapore; 3 Department of Orthopaedic Surgery, Sengkang General Hospital, Singapore; 4 Department of Orthopaedic Surgery, Centre for Orthopaedics, Singapore

**Keywords:** hallux mallet, extensor hallucis longus tendon, extensor hallucis longus

## Abstract

Injuries of the extensor hallucis longus (EHL) tendon are a rare phenomenon, with most occurring due to lacerations or penetrating injuries. Closed traumatic ruptures of the EHL are described as “Mallet injuries of the toe”. These can be classified as bony or soft mallet injuries depending on the presence or absence of a fracture at the insertion site of the EHL tendon in the distal phalanx. We present a case of a 33-year-old woman who presented with a hyperflexion injury to the left big toe with inability to extend the big toe. Ultrasound showed complete rupture of the EHL tendon with retraction proximal to the hallucal interphalangeal joint of the big toe. The patient was treated through transarticular pinning and repair using the Arthrex Mini Bio-Suture Tak with a 2-0 fibre wire. Six months post-operatively, the patient had symmetrical EHL power and full range of motion of the toe. The lessons to be drawn from this case report are that isolated hallux mallet injuries are rare and can be easily missed in the absence of penetrating wounds. Patients who have such injuries should be investigated early with the appropriate imaging techniques such as ultrasound or MRI and treated surgically.

## Introduction

The extensor hallucis longus (EHL) lies in the anterior compartment of the leg between the tibialis anterior and the extensor digitorum longus. It arises from the middle two-fourths of the medial surface of the fibula and from the adjacent anterior surface of the interosseous membrane. It primarily inserts over an oval footprint on the dorsal surface of the distal phalanx 2mm distal from the distal interphalangeal joint. At the metatarsophalangeal joint, a thin prolongation from each side of the tendon covers the dorsal surface of the joint. An expansion from the medial side of the tendon to the base of the proximal phalanx is also usually present^1^. This prevents any ruptures from proximally migrating beyond the metatarsophalangeal joint.

Injuries of the EHL tendon are a rare phenomenon, with most occurring due to lacerations or penetrating injuries. Closed traumatic ruptures of the EHL are described as “Mallet injuries of the toe”^2^. These can be classified as bony or soft mallet injuries depending on the presence or absence of a fracture at the insertion site of the EHL tendon in the distal phalanx.

## Case Report

**Fig 1:**
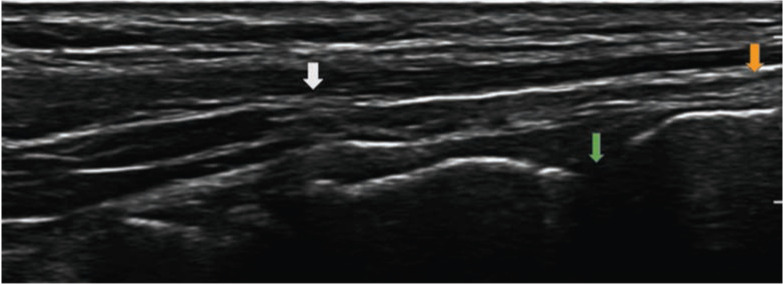
Ultrasound findings of the hallux. Tear in the extensor hallucis longus (EHL) tendon with retraction proximal to the interphalangeal joint up to the mid proximal phalanx (white arrow). Fluid gap at the dorsal lip of the left 1st distal phalangeal base where the EHL is expected to insert (orange arrow).

We present a case of a 33-year-old woman who presented to the emergency department with a sudden hyperflexion injury to the left big toe after falling down two steps on the same day. The patient had no diabetes, corticosteroid usage, or previous injections on the foot to cause any pathological tears. On examination, there was pain and ecchymosis on the left big toe. The patient was also unable to perform active extension of the hallux at the interphalangeal joint. The radiograph showed no fractures. An ultrasound of the affected region of the foot showed that at the dorsal lip of the left first distal phalangeal base, where the EHL is expected to insert, a fluid gap was noted, signifying a complete rupture of the tendon (Fig. 1). We offered surgical repair in view of extensor lag and inability of active extension of the interphalangeal joint of hallux.

**Fig. 2:**
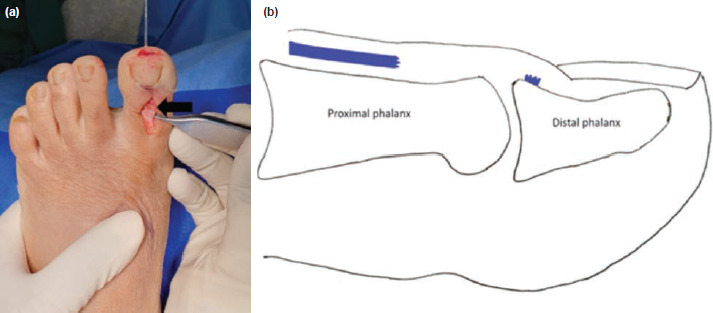
(a) Intra-operative photograph and (b) schematic image demonstrating the complete rupture of the EHL distally with retraction (arrow). The patient was followed-up four weeks later in the clinic for removal of the K-wire. At six months, the patient had symmetrical EHL power and range of motion across both feet. There was no pain and no nail deformities.

**Fig. 3:**
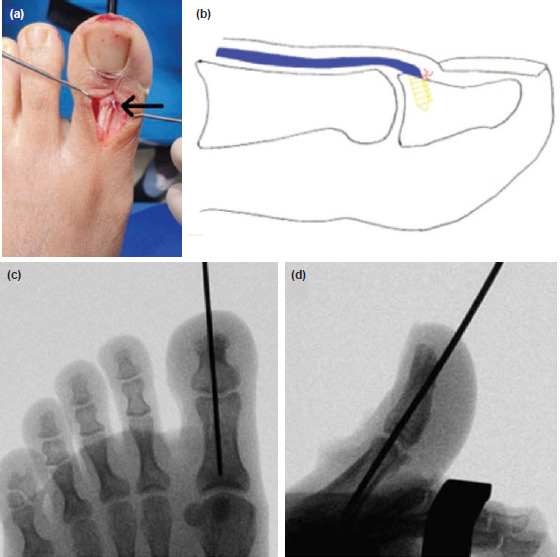
(a) Intra-operative photograph and (b) the schematic image showing the completed surgical repair of the EHL tendon (arrow). (c) Anterior-posterior and (d) lateral intra-operative radiographs showing the resting position of the big toe with retrograde pin inserted.

In the operation room, patient was placed supine on a radiolucent table and given regional anaesthesia. A percutaneous retrograde 1.6mm K-wire was passed from the distal phalanx into the proximal phalanx holding the toe in 10° dorsiflexion and confirmed under image intensifier. A dorsal midline incision was performed over the hallux which exposed the torn EHL with a 1cm retraction from the insertion point over the mid proximal phalanx, with clean edges (Fig. 2). The surgical repair required a double loaded fibre tape suture anchor [Arthrex Mini Bio-SutureTak 8.5mm x 2.4mm], which was inserted onto the footprint of EHL insertion, and the tendon end was repaired onto it (Fig. 3). K-wire position was reconfirmed after skin closure.

## Discussion

Given the rarity of isolated hallux mallet injuries, there has not been a consensus on how these injuries should be managed, with examples of both conservative and surgical management in current literature. The management of mallet finger injuries, which are more common and more well-established, has been used as a reference by several authors in the development of their regimens when managing isolated hallux mallet injuries^3^.

A review of literature only revealed 10 other cases of hallux mallet injuries, of which only one was a soft mallet injury. Most cases were bony injuries affecting the middle age group and all had a severe hyper plantarflexion injury. For the identified case of a soft mallet injury, there was a delay in presentation for more than a week in three cases, and due to the chronicity of the rupture and degree of retraction of the EHL, the surgical team decided to repair the injury with a suture anchor and K-wire transfixion. Prior to 2001, cases were treated with splints due to lack of surgical literature. These cases reported fair results but were not able to correct extensor lag. With improved surgical literature, EHL ruptures were subsequently treated with surgical repair, achieving good to excellent results including correction of the extensor lag. We have tabulated these cases for reference in Table I.

**Table I:** Review of Hallux Mallet injuries

No	Year of publication	Author	Bony (articular involvement)/Soft	Age/Sex	Time to presentation in days	Investigation	Treatment	F/U	Final result
1	2020	Current article	Soft	33/F	1	XR USG	Transarticular pinning and repair with Arthrex Mini Bio-SutureTak	6m	Excellent
2	1999	Rapoff *et al*^3^	Bony (45%)	32/M	2	XR	Dorsiflexion toe splint	4m	Fair
3	2001	Hennessey *et al*^2^	Bony (15%)	45/M	10	XR	Dorsal thermoplastic extension splint	2m	Fair
4	2007	Nakamura^3^	Bony (<10%)	51/M	1	XR	Transarticular pinning	12m	Good
5	2011	Wada *et al*^3^	Bony (50%)	49/M	1	XR CT	Extension block pinning	2m	Excellent
6	2013	Martin *et al*^3^	Bony (40%)	16/M	4	XR	Open reduction and k-wire fixation	6m	Excellent
7	2013	Hong CC *et al*4	Bony (40%)	39/F	7	XR	Fixation with suture anchor	18m	Excellent
8	2013	Hong CC *et al*^4^	Bony (30%)	46/M	1	XR	Fixation with suture anchor	18m	Excellent
9	2015	Kent *et al*^3^	Soft	13/F	21	XR USG	Repair with suture anchor and transarticular k-wire pinning	6m	Excellent
10	2019	Kawashima *et al*^3^	Bony (50%)	42/M	-	XR	Modified extension block technique	6m	Excellent
11	2020	Pierpaolo *et al*^3^	Bony (10%)	52/M	5	XR MRI	Fixation with Arthrex SwiveLock	2m	Excellent

Abbreviations: F/U: follow-up, No: number, B: bony, M: male, F: female, XR: radiograph, USG: ultrasonography, m: month

While Hong *et al* had previously described the technique of using a suture anchor without transarticular immobilisation of the interphalangeal joint^4^. We, however, felt that the tendon repair with the anchor should be protected with a K-wire to ensure correct alignment was maintained throughout the recovery of the injury. Hence, in this repair, it was imperative to insert the trans articular k-wire prior to anchor the repair to prevent loosening of the suture anchor. Furthermore, post-operatively our patient did not have any complications, and recovered with symmetrical EHL power and range of motion across both feet.

It is recommended that the sooner the treatment is performed after the injury, the simpler it is to reduce it successfully^5^. Hence, while MRI is the investigation of choice in the case of soft mallet injuries, we performed an ultrasound examination as it is cheap, easy to perform and more readily available. All the authors have also used radiographs of the foot as part of their initial investigations and can be useful in identifying avulsion fractures. However, in the case of a bony mallet, CT scanning would be useful in showing the details of the articular surface fracture.

A history of a hyper plantarflexion injury, clinical examination for the extensor lag of the interphalangeal joint and presence of a bony avulsion on a true lateral view can help in the diagnosis of avulsion fracture.

Pre-operatively, it is prudent to appreciate the distance of retraction. The tear cannot retract beyond the proximal phalanx because at the metatarsophalangeal joint, a thin prolongation from each side of the tendon that covers the dorsal surface of the joint. During exposure of the joint, the dissection must be kept proximal to the proximal nail fold to prevent germinal matrix injury and nail deformity.

The lessons to be drawn from this case report are that isolated hallux mallet injuries are rare and can be easily missed in the absence of penetrating wounds. Patients who have such injuries should be investigated early with the appropriate imaging techniques such as ultrasound or MRI and treated surgically.
